# Atrial giant cell myocarditis with preserved left ventricular function: a case report and literature review

**DOI:** 10.1186/s13019-023-02316-z

**Published:** 2023-07-14

**Authors:** Yilin Tang, Lin Qi, Ling Xu, Lei Lin, Junfeng Cai, Wei Shen, Yang Liu, Ming Li

**Affiliations:** 1 Department of Radiology, Huadong Hospital Affiliated to Fudan University, No. 221 West Yanan Road, Shanghai, 200040 China; 2 Department of Cardiovascular Surgery, Huadong Hospital Affiliated to Fudan University, Shanghai, China

**Keywords:** Giant cell myocarditis, Atrial myopathy, Atrial fibrillation

## Abstract

Giant cell myocarditis (GCM) is a rare and fatal inflammatory disorder induced by T-lymphocytes, typically affecting young adults. Generally, this disease presents with a rapidly progressive course and a very poor prognosis. In recent years, atrial GCM (aGCM) has been recognized as a clinicopathological entity distinct from classical GCM. As described by retrievable case reports, although its histopathological manifestations are highly similar to those of classical GCM, this entity is characterized by preserved left ventricular function and atrial arrhythmias, without ventricular arrhythmias. aGCM tends to show benign disease progression with a better clinical prognosis compared with the rapid course and poor prognosis of vGCM. We report a patient with aGCM with a history of renal abscess whose persistent myocardial injury considered to be associated with a history of renal abscess. Infection could be a potential trigger for the development of aGCM in this patient. An extensive literature review was also performed and the following three aspects were summarized: (1) Epidemiology and histopathological characteristics of aGCM; (2) The role of imaging in the evaluation of aGCM; (3) Diagnostic points and therapeutic decisions in aGCM.

## Introduction

Giant cell myocarditis (GCM) is a rare and fatal inflammatory disorder induced by T-lymphocytes, typically affecting young adults [1]. Generally, this disease presents with a rapidly progressive course and a very poor prognosis. GCM is characterized by acute heart failure, ventricular arrhythmias, and varying grades of atrioventricular block. Uncommon findings, including pericardial tamponade and atrial masses, have been previously reported in GCM cases. As its clinical presentation of overlap with other cardiac diseases, the imaging features are nonspecific, and due to its rarity, the timely diagnosis of GCM is challenging and the definite diagnosis relies on the endomyocardial biopsy (EMB). Although the underlying pathophysiological mechanisms of GCM remain elusive, statistics show that up to 20% of GCM cases are associated with other autoimmune diseases, indicating that altered immunity may be a causal factor [2]. In recent years, atrial GCM (aGCM) has been recognized as a clinicopathological entity distinct from classical GCM [3]. As described by retrievable case reports, although its histopathological manifestations are highly similar to those of classical GCM, this entity is characterized by preserved left ventricular function and atrial arrhythmias, without ventricular arrhythmias [3–10]. aGCM tends to show benign disease progression with a better clinical prognosis compared with the rapid course and poor prognosis of vGCM. The atrial myocardium is not routinely sampled at autopsy or during routine pathology, thus, knowledge of the atrial myocardium originates only from retrospective case descriptions, making its diagnosis challenging [7]. We report a patient with aGCM with a history of renal abscess whose persistent myocardial injury considered to be associated with a history of renal abscess. Infection could be a potential trigger for the development of aGCM in this patient. An extensive review was also performed and the following three aspects were summarized: (1) Epidemiology and histopathological characteristics of aGCM; (2) The role of imaging in the evaluation of aGCM; (3) Diagnostic points and therapeutic decisions in aGCM.

## Case presentation

A 39 year-old man was admitted to our hospital in January 2022 because of a week-long history of palpitations. In March 2018, the patient had been admitted to our hospital because of recurrent fever for one month and pyuria for ten days. Laboratory tests showed an increased level of neutrophils (84.2%, normal: 40–75%), decreased lymphocytes (11.5%, normal: 20–50%), decreased red blood cells (3.42 × 10^12^/L, normal: 4.0–5.5 × 10^12^/L), decreased hemoglobin (80 g/L, normal: 120–160 g/L), markedly increased C-reactive protein (CRP) (356.9 mg/L, normal: < 10 mg/L), elevated serum amyloid protein(139.6 mg/L, normal: < 10 mg/L), increased erythrocyte sedimentation rate (ESR) (80 mm/h, normal: < 20 mm/h), and elevated fibrinogen (7.42 g/L, normal: 2.0–4.5 g/L).; urine microscopy showed an increased leukocyte count (25–30/HP, normal: < 5/HP), while urine bacterial culture was negative. Computed tomography (CT) examination displayed massive bilateral perinephric exudate and swelling of the renal pelvis and calyx wall with both rough upper ureters. During the hospitalization, the patient had fever around 2–4 pm every day, with the highest temperature of 38.5 °C. We organized several expert consultations and made several revisions to the medication regimen. (1st: Paracetamol + Levofloxacin; 2nd: Imipenem + Amikacin; 3rd: Fluconazole + Meropenem + Fosfomycin), whereas the improvement was unremarkable. CRP level (99.3 mg/L) significantly decreased, while it was still higher than normal level. A subsequent urine culture revealed the growth of Candida tropicalis, the 4th modification was applied to the medication regimen (Linezolid + Fosfomycin). After that, the body temperature returned to normal and the urine culture was negative. After one week of stabilization, treatment was changed to Linezolid + Ofloxacin. The patient was subsequently discharged. As the kidney condition was complicated and the cardiac symptoms were not prominent at that time, no comprehensive cardiac examination was performed. Only an enlarged left atrium and a small amount of pericardial effusion were found on echocardiogram during admission, which was not taken seriously by the clinician. The patient's electrocardiogram (ECG) at admission and discharge were both normal.

**Fig. 1:**
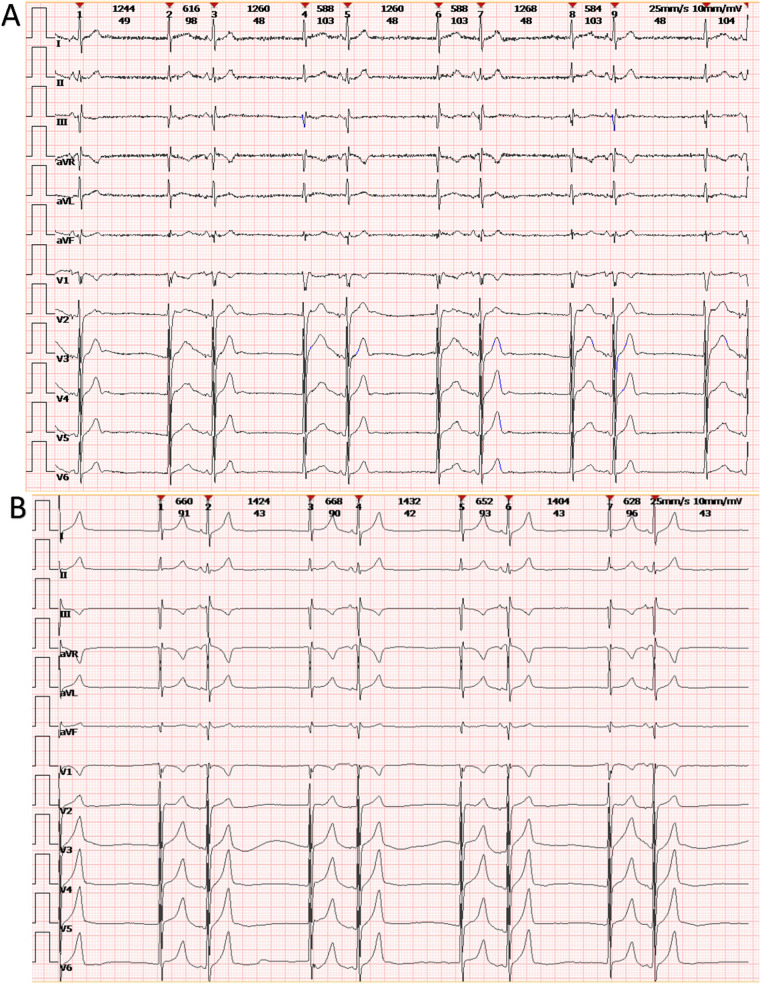

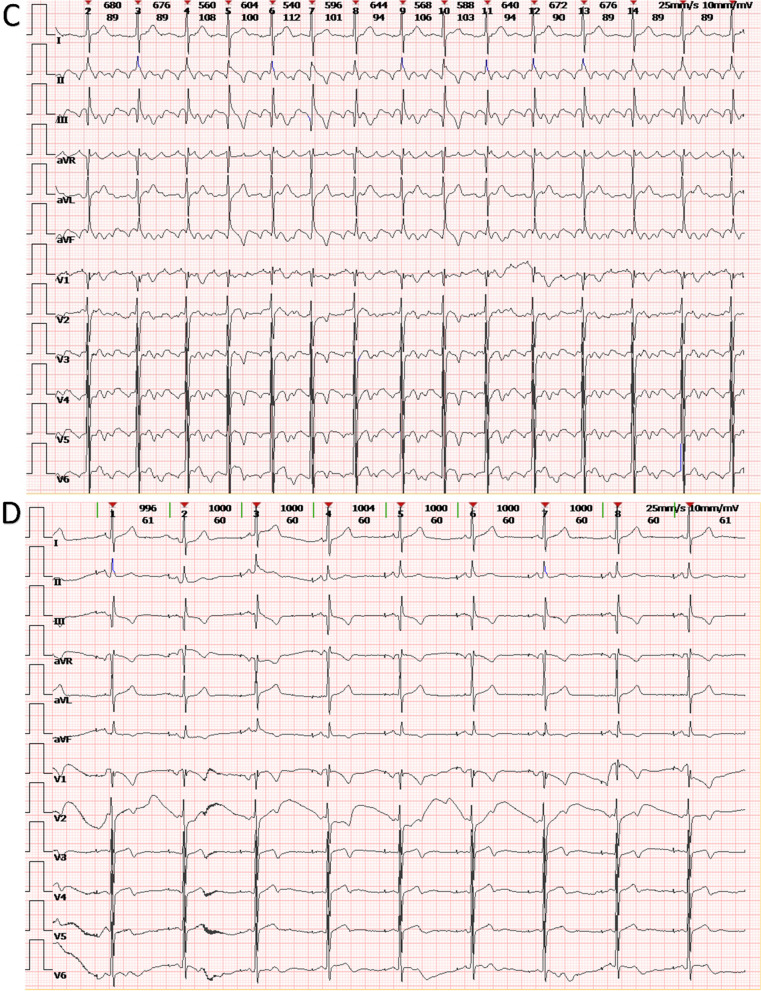
The ECGs of our case on the day of admission and on the morning of the day of cardiac tumor resection both demonstrated junctional escape-sinus capture bigeminy (**A** and **B**). After one week of pharmacological intervention following the temporary transvenous pacemaker implantation, the patient's ECG (**C**) showed the absence of P waves in all leads and the appearance of rapid and regular sawtooth-shaped F waves (250–350 bpm), predominantly with a 2:1 atrioventricular conduction ratio and occasional irregular f waves (350–450 bpm). The patient's ECG (**D**) after the implantation of a permanent pacemaker showed pacemaker-induced rhythm

**Fig. 2:**
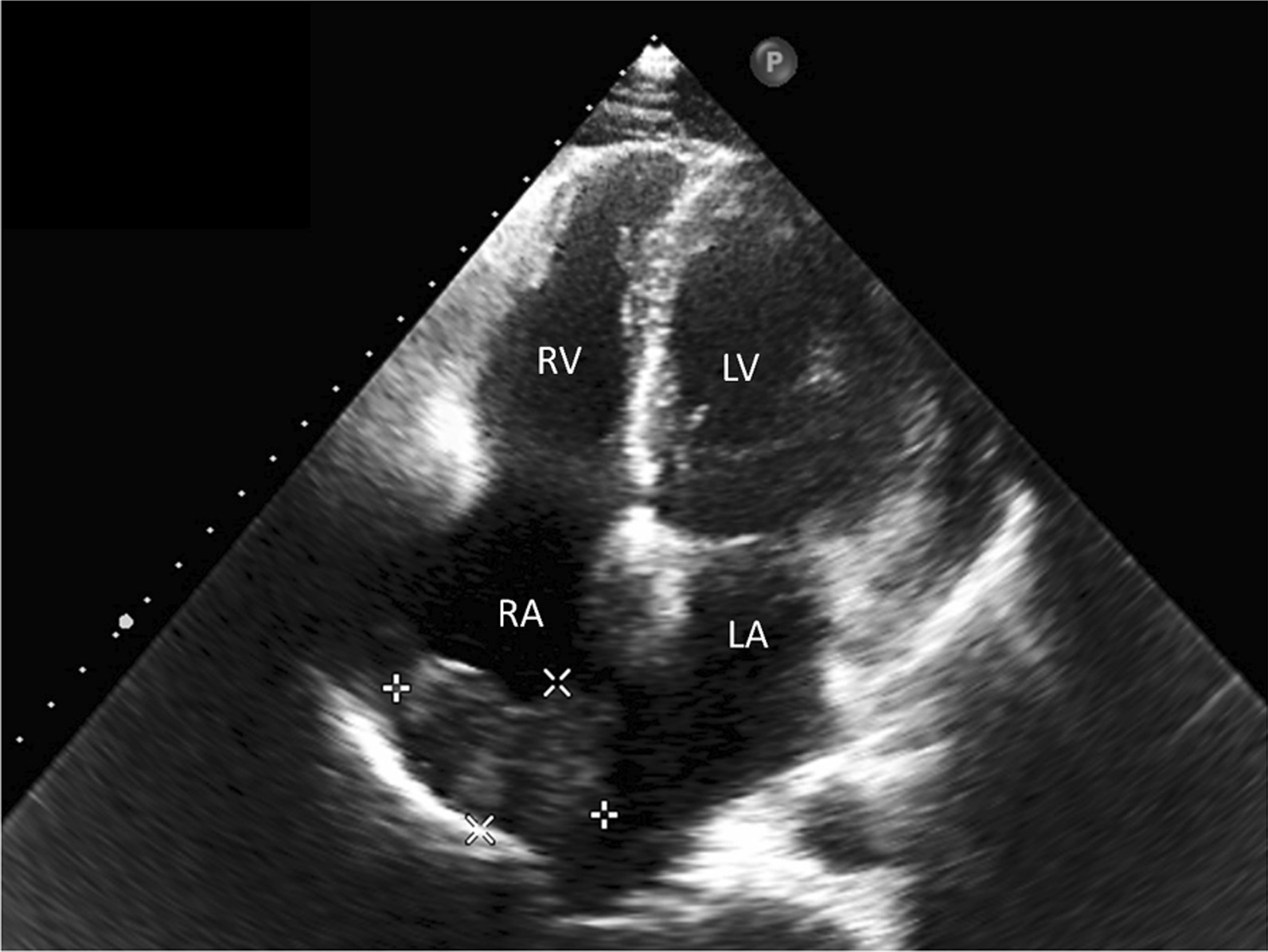
The echocardiogram revealed the presence of a moderately echogenic mass (white crosses) within the right atrium. The mass measures approximately 53 × 30 mm in size with an irregular morphology and relatively well-defined margins, displaying relatively uniform internal echo. *RV* Right ventricle, *LA* Left atrium, *RA* Right atrium, *LV* Left ventricle

**Fig. 3:**
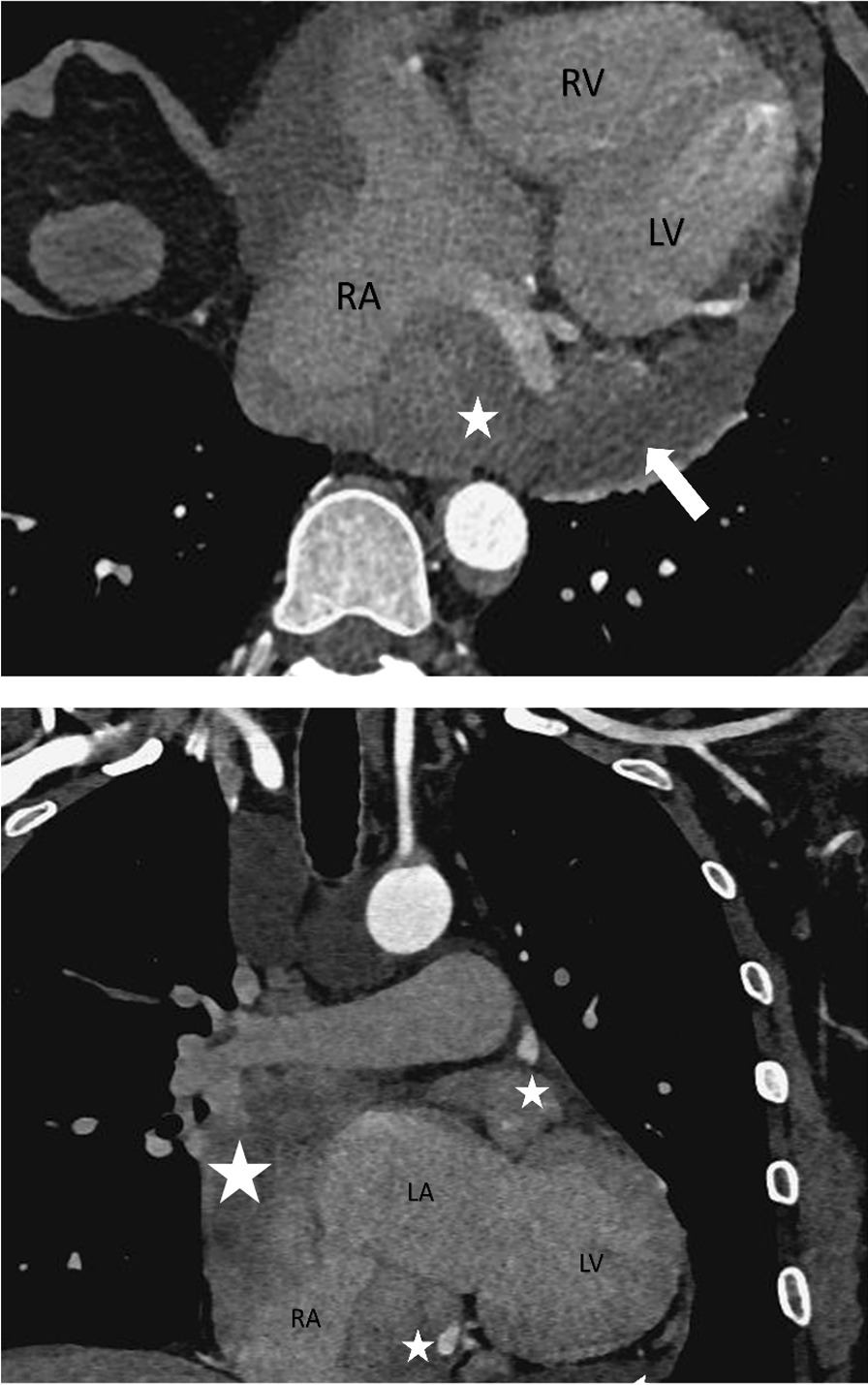
Both the axial and coronal CT images demonstrated anomalous soft tissue masses (pentagon) present in the right atrium, diffusely infiltrating and penetrating the atrial wall, resulting in the disappearance of the normal structure of the right atrium. Visible pericardial effusion (white arrow) was observed surrounding the heart. Abnormal nodules (pentagon) were observed around the left anterior descending coronary artery on the coronal image. *LA* Left atrium, *RA* Right atrium, *LV* Left ventricle

**Fig. 4:**
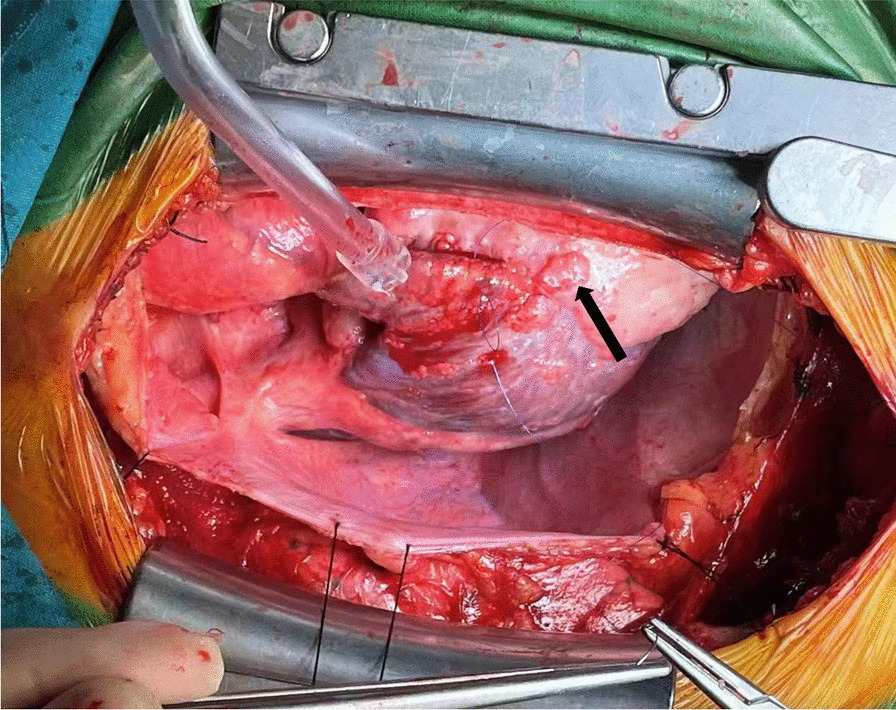
The surgeon opened the mediastinum and observed a nodular lesion on the outer wall of the right atrium (black arrow)

**Fig. 5:**
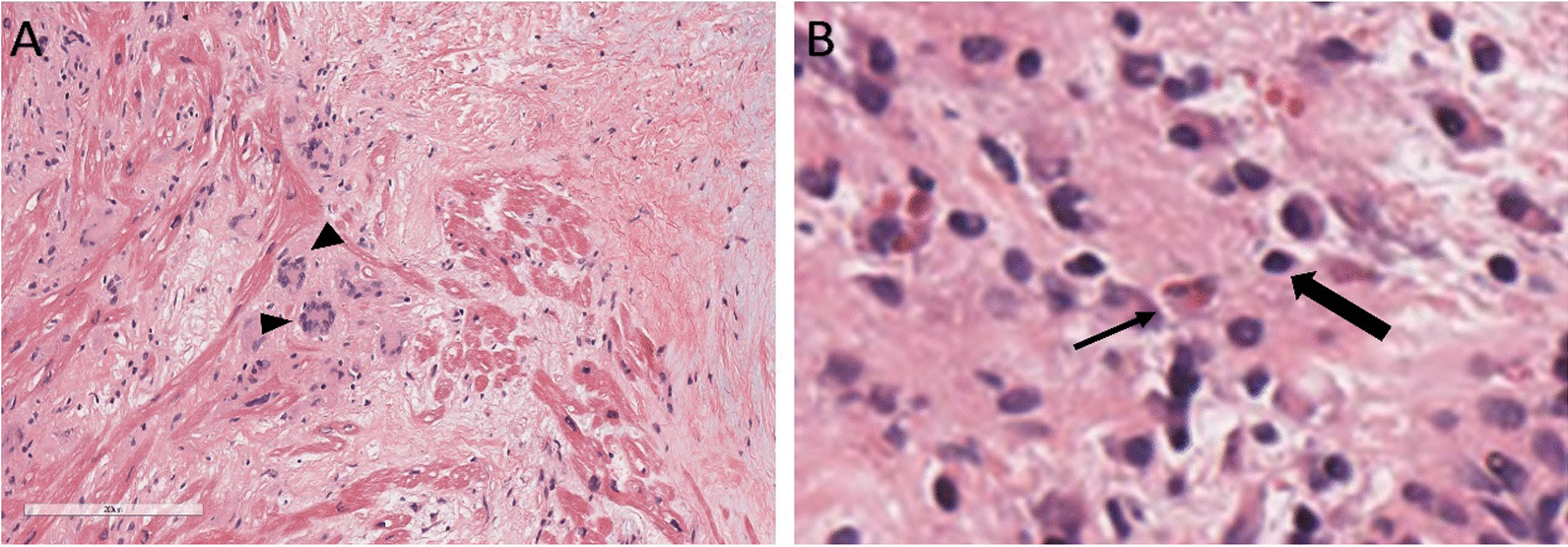
Histology of our case showed lymphocytic cells (thick arrow), eosinophils (thin arrow), multinucleated giant cells (black triangle) infiltration, and myocardial fibrosis (**A** and **B**)

In 2022, the patient was admitted to our hospital with cardiac symptoms. Tachycardia-bradycardia syndrome was detected on dynamic ECG. The ECG (Fig. 1A) revealed junctional escape-sinus capture bigeminy. Although the slowest heart rate was not captured on this ECG, 24 h bedside monitoring revealed a tachycardia-bradycardia syndrome, with a heart rate of 140 beats/minute during atrial flutter and a heart rate of only 40 beats/minute during bradycardia. Based on the patient's clinical symptoms and electrocardiographic abnormalities, the diagnosis of sick sinus node syndrome (SSS) was considered. The echocardiogram (Fig. 2) revealed a space-occupying lesion with moderate echogenicity located in the right atrium, measuring approximately 55 × 30 mm in size, bi-atrial enlargement, tricuspid regurgitation (mild-moderate), and a small amount of fluid in the pericardial cavity, while left ventricular ejection fraction (67%), and segmental motion of the ventricular wall were both normal. Cardiac CT (CCT) revealed multiple nodules and masses in the right atrial wall, peri-coronary artery, and left side of the inferior vena cava (Fig. 3). CRP (27.3 mg/L, normal; < 10 mg/L), ESR (40 mm/h; normal: < 20 mm/h),Serum B-type natriuretic peptide (133 pg/ml; normal < 100 pg/ml), fibrinogen (6.25 g/L; normal 2–4 g/L), immunoglobulin G (IgG)-LAMBDA light chain (2.73 g/L; normal 0.9–2.1 g/L), KAPPA light chain (5.20 g/L; normal 1.7–3.7 g/L), and IgG4 (2.545 g/L; normal 0.03–2 g/L) levels were elevated. No clinical evidence of other autoimmune diseases was found. Moreover, examinations of potentially pathogenic agents, including antibodies to syphilis spirochetes, human immunodeficiency virus, hepatitis A, B, and C viruses, and mycobacterium tuberculosis, were all negative. The results of autoimmune antibody testing demonstrated negative for the following antibodies: anti-Scl-70 antibody, anti-mitochondrial antibody, anti-PCNA antibody, anti-nucleolar antibody, anti-Jo-1 antibody, anti-histone antibody, anti-U1-nRNP antibody, anti-centromere antibody, anti-SS-A antibody, anti-SS-B antibody, and anti-Sm antibody. The levels of anti-smooth muscle antibody (9.90 U, normal: 0–22 U), anti-nuclear antibody (11.1 U/ml, normal: 0–55 U/ml), anti-double stranded DNA antibody (23.95 IU/ml, normal: ≤ 200 IU/ml), anti-glomerular basement membrane antibody (2.4 Units, normal: 0–20 Units), and anti-intrinsic factor antibody (4.4 Units, normal: 0–22 Units) were all within the normal range. Due to the friable texture of the inflammatory mass, we were concerned that the mass could easily fragment after EMB and lead to severe complications such as embolism. Furthermore, the size of the right atrial mass made it difficult to pass through the tricuspid valve, increasing the risk of injury and embolism. Therefore, we opted for cardiac tumor resection instead of EMB. Other than aGCM, our suspected diagnoses of this patient before surgery included atrial myxoma, atrial malignant tumor (primary or metastatic), and IgG4 related disease (Table 1). Four days after admission, cardiac tumor resection was conducted (Fig. 4). However, during the surgery, we found that the lesion was scattered and multifocal, making it impossible to remove completely. Therefore, only a biopsy was performed to remove the atrial mass and part of the pericardium by wrapping the lesion tissue on the surface of the right atrium. The histopathological examination (Fig. 5) of resected tissue (size: 0.8 × 0.8 × 0.4 cm^3^) revealed large areas of myocyte replacement by hyperplastic fibrous tissues and inflammatory cell infiltrate composed of T-lymphocytes, plasma cells, eosinophils, and scattered multinucleated giant cells, which was consistent with the pathological features of GCM. Immunohistochemical results documented the lymphocytic infiltrate with CD3+ and CD5+ T lymphocytes, fewer CD20+ B lymphocytes, scattered CD68+ histiocytes and giant cells, without IgG4+ plasma cells. No bacteria, such as acid-resistant bacilli, fungi, or other parasites were found, and no foreign body was detected. After the surgery, the patient developed atrial flutter (140–180 beats/minute), accompanied by bradycardia (38 beats/minute). In addition, the patient had a symptom of chest tightness. Due to the complexity of the condition, temporary transvenous pacing (VVI mode) was performed to maintain the heart rate. Considering that the patient may benefit from anti-inflammatory treatment, he received continuous intravenous infusion of methylprednisolone for five days (80 mg daily), followed by a combination of methylprednisolone (40 mg daily) and cyclosporine (50 mg twice daily). However, after ten days of pharmacological intervention, there was no improvement in the arrhythmia, and thus a dual-chamber permanent pacemaker (DDD mode) was implanted. After permanent pacemaker implantation, the patient was treated with immunosuppressive therapy including methylprednisolone (24 mg once daily) and cyclosporine (50 mg twice daily). At outpatient follow-up one month after starting the immunosuppressive therapy, the patient reported significant symptom relief. Subsequently, the dosages of methylprednisolone and cyclosporine were gradually reduced. The total duration of immunosuppressive therapy after permanent pacemaker implantation was six months. At present, the patient continues to do well.

**Table 1:** Suspected preoperative diagnosis of this patient and the basis for the final exclusion of these diagnoses

Suspected diagnoses	Exclusion reasons
Atrial myxoma	Atrial myxomas are mostly located in the left atrium, and most have a pedicle attached to the atrial septum or left atrial wall. They have good activity and move with the heart cycle [11]. But the patient's echocardiography showed that the mass was located in the right atrium, with a fixed position. Combined with the ultrasound findings and the location of the mass, it was not like a typical atrial myxoma
Primary cardiac angiosarcoma	Primary cardiac angiosarcoma is a rare malignant tumor that originates in the heart, with the majority of cases arising from the right atrium [12]. Typically presenting as a large mass, it has a tendency to invade adjacent tissues and can easily metastasize to the lungs, liver, and brain in the early stage [13]. Given that the mass in this patient was localized to the right atrium and there was no indication of distant metastasis, insufficient diagnostic evidence existed to support a diagnosis of angiosarcoma
Atrial metastatic tumors	Based on our clinical experience, the majority of tumors that metastasize to the heart are located in the right atrium, with poor activity and irregular morphology. In this patient, normal levels of tumor markers were observed in blood tests, and no signs of tumors were found in abdominal ultrasonography or head CT scans, indicating no evidence of malignant tumor metastasis
IgG4-related disease	The patient's CCT showed multiple nodules surrounding each coronary artery and on the left side of the inferior vena cava. Additionally, the serum IgG4 level was mildly elevated. Therefore, we considered IgG4 related disease before surgery and ultimately ruled out this disease through pathological examination

## Epidemiology

GCM is an uncommon disorder that was first explored in 1905 [14]. According to autopsy reports from Japan, the United Kingdom, and India, its incidence ranges from 0.007 to 0.051% [15, 16]. Compared with typical ventricular GCM (vGCM), aGCM is a rarer pathological entity of GCM that mainly affects the atria. aGCM was first reported by McCrea in 1964 [9]. The first reported aGCM patient had a history of rheumatic fever and rheumatic mitral valve disease. aGCM was regarded as an incidental finding during his elective mitral valve replacement. As no evidence of infection was found in this patient and no typical rheumatic lesions were present in his left auricle lesion, he was diagnosed with chronic GCM of unknown origin. To date, the lack of familiarity still exists in the current knowledge regarding this entity. We retrieved a total of 15 aGCM patients (median age of 53 years,7 male cases) on PubMed and Medline online databases, of whom 9 cases were discovered unexpectedly during elective valve surgery, 1 case was discovered unexpectedly during coronary artery bypass grafting, 4 cases with acute heart failure(HF) underwent diagnostic atrial biopsy, and 1 case died of other causes and his asymptomatic aGCM lesions were found incidentally at autopsy [3–10]. The above findings suggest that aGCM is more common than we thought.

## Pathogenesis and histopathology

Multiple circulating autoantibodies, some of which are cardiac-specific, have shown to play a key role in the progression and induction of myocarditis [17, 18]. These autoantibodies are directed against different autoantigens that are mainly unrestricted when exposed to the myocardium. However, in the presence of immune abnormalities, these autoantigens are attacked by autoantibodies, leading to autoimmune diseases. The exact mechanisms of how these autoantibodies persist or even induce cardiac-specific autoimmune responses have not been fully understood. Endogenous (immunocompromised) or exogenous factors (infections), molecular mimicry and cross-reactivity may play an important role in the induction of cardiac autoimmune responses [3, 19, 20]. aGCM and vGCM have overlapping pathogenesis, both are autoimmune diseases, and have genetic susceptibility. However, aGCM is distinguished from typical vGCM, and the presence of atrial-specific antigens may be involved in the development of aGCM [3, 21].

Although aGCM more or less involves the ventricles, it mainly affects the atria. The most common sites of aGCM are the left atrial appendage and the left atrium, followed by the right atrial appendage, the right atrium, and the walls of the blood vessels surrounding the heart, while a single site is not mainly involved [3–5, 10, 22]. T lymphocytes and characteristic multinucleated giant cells infiltrating the atria are the histopathological features of aGCM [23]. T lymphocytes mainly include CD3+ and CD8+ T lymphocytes. Additional inflammatory cells include eosinophils, plasma cells, B cells, and histiocytes. aGCM in the acute inflammatory phase can cause myocardial hypertrophy and myocardial necrosis [7]. As persistent myocardial inflammation in aGCM moves from the acute to the chronic phase or further develops into chronic inflammatory cardiomyopathy, histopathological examination may show less inflammatory infiltrates, primarily fibrosis [23]. Reduced atrial systolic function, poor blood return, abnormal valve function, and atrial arrhythmias in aGCM promote the formation of appendage thrombus.

## Non-invasive examination techniques

### Echocardiography

Due to its wide availability and affordability, echocardiography represents the first-line cardiac imaging modality [24]. Echocardiography plays an important role in the initial diagnosis of aGCM, and transesophageal echocardiography can improve the sensitivity of detecting cardiac abnormalities in aGCM [25]. Almost all patients with aGCM have abnormal echocardiographic findings. Echocardiographic findings in aGCM include atrial dilatation (bilateral/unilateral), atrial hypokinesis, appendicular thrombus, mitral/tricuspid stenosis/regurgitation, atrial wall thickening due to inflammatory infiltration, and atrial edema [3, 8, 14]. Because aGCM involves more or less ventricles, edema and inflammatory infiltration of the ventricular wall may lead to wall thickening; the continued evolution of the inflammatory process may result in ventricular enlargement. Several published case reports have also identified the above ventricular changes in aGCM [3, 6]. It is noteworthy that ventricular ejection fraction and ventricular motility were normal in all published cases and our case. Atrial masses are less common and may be an atypical manifestation of aGCM. Atrial masses were found in only two cases (including our case), both of which were rich in fibrous tissue composition [8]. Echocardiography is helpful in the differential diagnosis of aGCM with atrial mass and atrial myxoma [26].

### Electrocardiogram

ECG is mainly used to reflect atrial arrhythmias and sinus node dysfunction in aGCM. Based on available case reports of aGCM, atrial arrhythmias in aGCM include atrial fibrillation and atrial flutter. Atrial arrhythmias in patients with aGCM are mostly persistent or chronic, but they can also be paroxysmal [27]. When aGCM involves the sinus node and its adjacent tissues, it causes them to undergo pathological changes leading to SSS [3, 5]. The manifestations of SSS include sinus bradycardia, ectopic atrial bradycardia, sinus node outlet block, sinus arrest, sinus node block, bradycardia arrhythmia syndrome, variable time insufficiency, and rhythm separation [28, 29]. In addition, about 50% of patients with SSS intermittently develop supraventricular tachycardia, called tachycardia-bradycardia syndrome, which is a typical ECG manifestation of SSS [29]. The aforementioned arrhythmias may not cause symptoms or exhibit only mild symptoms [30].

### Cardiac magnetic resonance imaging

Cardiac Magnetic Resonance Imaging (CMRI) is the preferred non-invasive imaging tool for the diagnosis of suspected myocarditis, providing essential information on the morphology and function of cardiac structures, and it has a unique potential for myocardial tissue characterization [31]. Myocardial edema, congestion, and capillary leakage during early gadolinium enhancement and myocardial cell necrosis and fibrosis during late gadolinium enhancement (LGE) are considered as the main CMRI manifestations of myocardial inflammation [31].

Atrial lesions in aGCM may be detected by abnormal high signal intensity of the atria on LGE, T2-weighted edema images and T1-weighted sequences (early enhancement before and after contrast injection). CMRI facilitates identification of patients who may require atrial EMB [21]. However, CMRI cannot establish the etiology of myocarditis and therefore cannot replace EMB for diagnostic testing of aGCM [20]. Furthermore, the frequent arrhythmias and chronic inflammation in aGCM may limit the diagnostic value of CMRI [32].

### Cardiac CT

CCT is a robust technique for structural and functional assessment of the heart [33, 34]. It possesses the advantages of operator’s independence, high spatial resolution, high temporal resolution, and additional anatomic views. Thus, CCT can accurately demonstrate the anatomy of the heart, coronary arteries, and great vessels. In addition, it can be applied to the evaluation of pericardial and cardiac masses [35].

The clinical presentation of aGCM may overlap with acute coronary syndrome (ACS, a consequence of coronary artery disease or vascular disease), and ECG and myocardial damage indices (e.g., cardiac troponin T or I, cTnT or cTnI) may also show findings comparable to those of “true” acute coronary syndrome [17]. As ACS is extremely prevalent and possesses a higher level of clinical suspicion, the etiology of ACS-like symptoms needs to be clarified before diagnosing aGCM. CCT has a high accuracy in diagnosing and excluding coronary artery disease (CAD) and enables clinicians to clarify the etiology of ASC [36, 37]. However, it is noteworthy that patients with aGCM may concurrently have CAD [3]. In addition, CCT of aGCM is not associated with bilateral pulmonary lymph nodes or right paratracheal lymph node enlargement, thus, CCT facilitates exclusion of cardiac sarcoidosis [38]. CCT has high spatial and temporal resolution and clearly shows the contours, lobulated shape, smooth surface, and narrow basal attachment to the atria of primary cardiac tumors (e.g., cardiac myxoma), a presentation that is significantly different from myocarditis and is associated with infiltrative damage to myocardial tissue [39]. Thus, CCT is advantageous to distinguish primary cardiac tumors from aGCM in the presence of atrial masses. However, similar to CMRI, CCT can only be used as an ancillary test and cannot be replaced with EMB in the diagnostic testing of aGCM [31, 40].

### Radionuclide imaging

^18^F-Fluorodeoxyglucose (^18^F-FDG) is an analog of glucose and can be used to identify tissues with high rates of glucose metabolism. ^18^F-FDG positron emission tomography (FDG-PET) uses the principle of increased glucose uptake by inflamed tissues to detect metabolic information in persistent myocardial inflammation [41]. Therefore, Atrial involvement in aGCM can be diagnosed on the basis of atrial ^18^F-FDG accumulation using FDG-PET. It is not typically used as a routine test for detecting myocarditis due to the availability of alternative imaging modalities (e.g., CMRI), the need for complex dietary modifications prior to the test, and the high radiation exposure [42]. However, it can be used as an alternative non-invasive diagnostic tool to CMRI in patients with contraindications to CMRI. Besides, FDG-PET can show high ^18^F-FDG uptake at different anatomical sites and therefore differentiate other autoimmune diseases with cardiac involvement (e.g., cardiac sarcoidosis, IgG4-related disease, and polymyalgia rheumatic) from aGCM [43–45]. In addition, it has been suggested that FDG-PET may be able to compensate for the lower diagnostic accuracy of CMRI in the chronic phase of aGCM [46].

## Diagnosis

Diagnostic criteria for clinically suspected myocarditis could be summarized as follows [20]:

1. ≥ 1 clinical presentation: cardiac symptoms, such as dyspnea, palpitations, syncope, chest pain; history of asthma and extracardiac autoimmune diseases; toxic substances; family history of dilated cardiomyopathy or myocarditis, etc.
2. ≥ 1 diagnostic criteria from different categories: elevated troponin levels; abnormal ECG findings; abnormal cardiac imaging findings, such as CCT, echocardiography, and CMRI; and abnormal myocardial tissue metabolism.

EMB is the only available method to confirm the diagnosis of myocarditis [47]. However, during EMB procedures, atrial tissue is not routinely sampled, which, together with inflammatory damage in aGCM more or less involving the ventricles, makes it difficult to distinguish aGCM from vGCM [21]. Therefore, Atrial tissue sampling should be performed in patients with suspected myocarditis who have clinical features of aGCM.

Based on the available case reports (including our case), we conclude that the diagnosis of aGCM requires evidence of myocardial pathology and clinical signs. Patients with typical myocardial pathological evidence of preserved left ventricular function with atrial arrhythmias or SSS should be diagnosed with aGCM, whereas those with ventricular HF with decreased ejection function cannot be diagnosed with aGCM.

## Treatment

The aim of treatment is to reduce cardiac symptoms, in order to improve patients’ quality of life. The treatment of aGCM includes the following aspects:

1. Symptomatic treatment: Symptomatic and supportive care according to guidelines for HF, atrial arrhythmias, sinus node abnormalities or comorbidities in patients with aGCM [48, 49]. Routine anticoagulation in patients who are at the risk of stroke (e.g., hypertension and diabetes) with combined AF/atrial flutter [50]. Warfarin and non-vitamin K oral anticoagulants (NOACs) are the main drug therapies for stroke prevention [51, 52]. However, NOACs are contraindicated in patients with mechanical heart valves, and their efficacy and safety in patients with rheumatic mitral stenosis are still under investigation [53]. Therefore, warfarin is currently recommended explicitly for these two groups of patients [54–56]. For symptomatic SSS and documented abnormal sinus node function with bradycardia, placement of a permanent pacemaker is recommended, and it is the most effective treatment to alleviate the symptoms of SSS [57].
2. Myocarditis-specific treatment: As with other types of myocarditis, aGCM can be the result of multiple causes and treatment is therefore multidisciplinary [58]. aGCM myocarditis treatment teams should always include cardiologists, radiologists, cardiovascular pathologists, clinical immunologists or rheumatologists, and infectious disease specialists.
3. Immunosuppressive therapy: Immunosuppression is used to treat the autoimmune type of myocarditis, which is based on the suppression of the cellular and humoral immunity to suppress myocarditis [32]. The main drugs used in immunosuppressive therapy include steroids (prednisone and prednisone), cyclosporine, and azathioprine, in which they most of which are mainly used in combination [59]. In the published literature, patients with aGCM showed a significant improvement in clinical symptoms and cardiac lesions after treatment with immunosuppressive therapy. However, there exists a subset of patients with aGCM who have achieved symptomatic relief with aggressive symptomatic and supportive treatment without immunosuppressive therapy, and these patients returned to normal life with no adverse effects at long-term follow-up [3, 8, 22]. Thus, the benefits of immunosuppressive therapy for aGCM need to be evaluated in specific clinical settings.

## Conclusion

It is broadly accepted that aGCM is a rare disease with a very low prevalence, whereas its true incidence appears to be greatly underestimated based on the lack of non-invasive and highly specific diagnostic methods, the typical overlap of symptoms with other more common/occasional cardiovascular diseases, and the fact that atria are not routinely sampled in EMB.

Autoimmune diseases, infections, genetic susceptibility, immune depression, molecular mimicry, and cross-reactivity may be risk factors for the triggering and development of aGCM. The case reported in the present study also indicated that aGCM is triggered and developed by fusion of endogenous and environmental (infectious) factors. However, our knowledge about aGCM is based on retrospective case reports and the lack of clinical and experimental animal models is noteworthy, which makes clarifying the specific triggers and causative factors for aGCM progression remains a challenge. With the development of imaging techniques, the integration of echocardiography, ECG, CCT, CMRI, and nuclear imaging can facilitate understanding the histopathological features of aGCM, thus helping to make diagnostic and therapeutic decisions. Nevertheless, EMB remains the diagnostic gold standard for etiology and differential diagnosis. Therefore, atrial tissue sampling should be performed during EMB procedures in highly suspected patients with aGCM. The therapeutic knowledge suggests that timely symptomatic and supportive treatment is effective for aGCM, while the recommendation of immunosuppressive therapy for aGCM should be assessed in specific clinical settings. As a pathological entity distinct from classical vGCM, aGCM has a relatively slow progression and good outcome without directly leading to fulminant death. In addition, atrial-specific antigens may be closely associated with triggering and maintaining the aGCM-specific autoimmune process, while further research is required to determine which antigens play an important role in the pathogenic mechanism.

## Data Availability

The data of our case that support the findings of this study are available on request from the corresponding author. The data are not publicly available due to privacy or ethical restrictions.
